# Label-free analysis of the characteristics of a single cell trapped by acoustic tweezers

**DOI:** 10.1038/s41598-017-14572-w

**Published:** 2017-10-26

**Authors:** Min Gon Kim, Jinhyoung Park, Hae Gyun Lim, Sangpil Yoon, Changyang Lee, Jin Ho Chang, K. Kirk Shung

**Affiliations:** 1 Department of Biomedical Engineering, University of Southern California, Los Angeles, CA 90089 USA; 2 Department of Biomedical Engineering, Sungkyunkwan University, Suwon, Gyeonggi-do 16419 Republic of Korea; 3 Department of Biomedical Engineering, Sogang University, Seoul, 04107 Republic of Korea; 4 Department of Electronic Engineering, Sogang University, Seoul, 04107 Republic of Korea

## Abstract

Single-cell analysis is essential to understand the physical and functional characteristics of cells. The basic knowledge of these characteristics is important to elucidate the unique features of various cells and causative factors of diseases and determine the most effective treatments for diseases. Recently, acoustic tweezers based on tightly focused ultrasound microbeam have attracted considerable attention owing to their capability to grab and separate a single cell from a heterogeneous cell sample and to measure its physical cell properties. However, the measurement cannot be performed while trapping the target cell, because the current method uses long ultrasound pulses for grabbing one cell and short pulses for interrogating the target cell. In this paper, we demonstrate that short ultrasound pulses can be used for generating acoustic trapping force comparable to that with long pulses by adjusting the pulse repetition frequency (PRF). This enables us to capture a single cell and measure its physical properties simultaneously. Furthermore, it is shown that short ultrasound pulses at a PRF of 167 kHz can trap and separate either one red blood cell or one prostate cancer cell and facilitate the simultaneous measurement of its integrated backscattering coefficient related to the cell size and mechanical properties.

## Introduction

The basic knowledge of physical and functional characteristics of cells is essential for understanding the unique features of various cells and the causative factors of diseases and determining the most effective treatments for diseases. Precise cell manipulation techniques have played a pivotal role in expanding the knowledge such as the molecular dynamics of living cells^1,2^, cell signalling pathways and networks^3,4^, and gene expression profiles^5,6^. Moreover, cell manipulation techniques can be used for discovering and developing new drugs^7,8^. For precise cell analysis, it is essential to identify and extract the same type of cells from a heterogeneous cell sample; otherwise, misleading information would be obtained^9–12^. For this reason, single-cell analysis techniques are preferable and have been developed for investigating various cellular behaviours among individual cells at the single-cell level.

Single-cell analysis requires cell sorting technologies that are categorized into label-aided and label-free methods. As label-aided methods, fluorescent-activated cell sorting (FACS)^13–15^ and magnetic-activated cell sorting (MACS)^16,17^ have been widely used for identifying and collecting cells of interest because they can provide rapid and reliable information about the target cells in a heterogeneous cell population. These capabilities facilitate fast and accurate separation of a large number of cells. However, cell labelling is labour intensive and time consuming in sample preparation. Additionally, fluorescent dyes tagged for FACS and particular antibodies for MACS may influence normal cellular physiology and functions^18,19^. For these reasons, label-free single-cell analysis techniques have attracted considerable attention because the complexity of sample preparation and analysis procedures is relatively low and intrinsic physical cell properties such as cell size, shape, compressibility, and polarizability can be measured while minimizing the effect on cell physiology and function^19–21^.

As a contact-free method, optical tweezers and optical stretcher were developed for trapping and deforming micron-sized particles and cells, respectively, by using single beam and double beam lasers. However, those methods exhibit not only low throughput, but also high susceptibility to alignment for laser radiations, heating, and photodamaging effects, which may cause irreversible cell membrane damage^22,23^. On the other hand, microfluidic systems have been used for label-free, high-throughput, and cost-effective single-cell analysis and have the advantage of analysing rare cells (e.g. circulating tumour cells). While heterogeneous cells are running through micro-channel networks in a microfluidic system, a physical source including dielectrophoretic forces^24,25^, laser radiations^26,27^, and standing surface acoustic waves^28–30^ is utilized for separating the target cells. To use the physical sources, however, various difficulties should be overcome, such as the fabrication of complex microelectrode for dielectrophoretic forces, expensive and sophisticated setup for laser radiations, and complicated alignment of standing surface acoustic waves. Otherwise, it is likely to reduce cell separation performance. Furthermore, this method frequently suffers from unexpected adverse effect on cell behaviour and response owing to uncoordinated shear stress and clogging in geometric microstructures^31,32^. After cell sorting in microfluidic systems, additional processing may be required to eliminate unwanted cells from the sorted group of cells, manipulate a single cell, and measure the physical and functional characteristics of a single cell.

As another label-free single-cell analysis technique, it was demonstrated that an acoustic tweezer exhibits the ability to grab a single cell or measure physical cell properties such as size, stiffness, and backscattering coefficient^33–35^. This device uses an acoustic microbeam produced by a tightly focused high-frequency ultrasonic transducer to capture a single cell. Moreover, it has a relatively simple and cost-effective system configuration compared to laser-based approaches. The acoustic tweezers transmit long ultrasound pulses to grab a single cell and subsequently short pulses to interrogate the target cell. It should be noted that long ultrasound pulses are used for securing sufficient acoustic intensity to capture a single cell^36^, and the capture and interrogation are performed using either the same transducer or different transducers in each processing. Therefore, it is difficult to hold the target cell while measuring the cell, which may lead to inaccurate measurement results. Additionally, the delivery of long ultrasound pulses may degrade cell viability because excessive acoustic energy possibly damages cells. To avoid these problems, theoretically, we hypothesize that short ultrasound pulses generated at a high pulse repetition frequency (PRF) can be used if a high-frequency, high-PRF ultrasound generator is available. This is because the acoustic intensity that determines the cell trapping force is proportional to both pulse length and PRF.

In this paper, we ascertain that the acoustic trapping force can be adjusted by the PRF rather than the pulse length. This enables us to capture a single cell, measure its physical properties simultaneously, and translate the measured cell to the target location for separation, because the echoes of short ultrasound pulses transmitted to trap a single cell can be used for measurements. For this purpose, we developed a tightly focused 153-MHz ultrasound transducer (i.e. a wavelength of 10 μm) and a low noise custom-built all-in-one front-end system. In particular, this front-end system exhibits the ability to generate short pulses at a maximum PRF of 1 MHz and to detect weak backscattering signals from a single cell. Moreover, an impedance matching network was developed to efficiently deliver electronic signals to the transducer and used to connect the transducer and the front-end system. The developed system was utilized for verifying the capability to identify the red blood cells (RBCs) and cancer cells (i.e. normal SV40 immortalized epithelial prostate: PNT1A) by measuring their integrated backscattering (IB) coefficients while trapping one of those cells with short ultrasound pulses.

## Results

### Trapping force controlled by PRF

**Figure 1:**
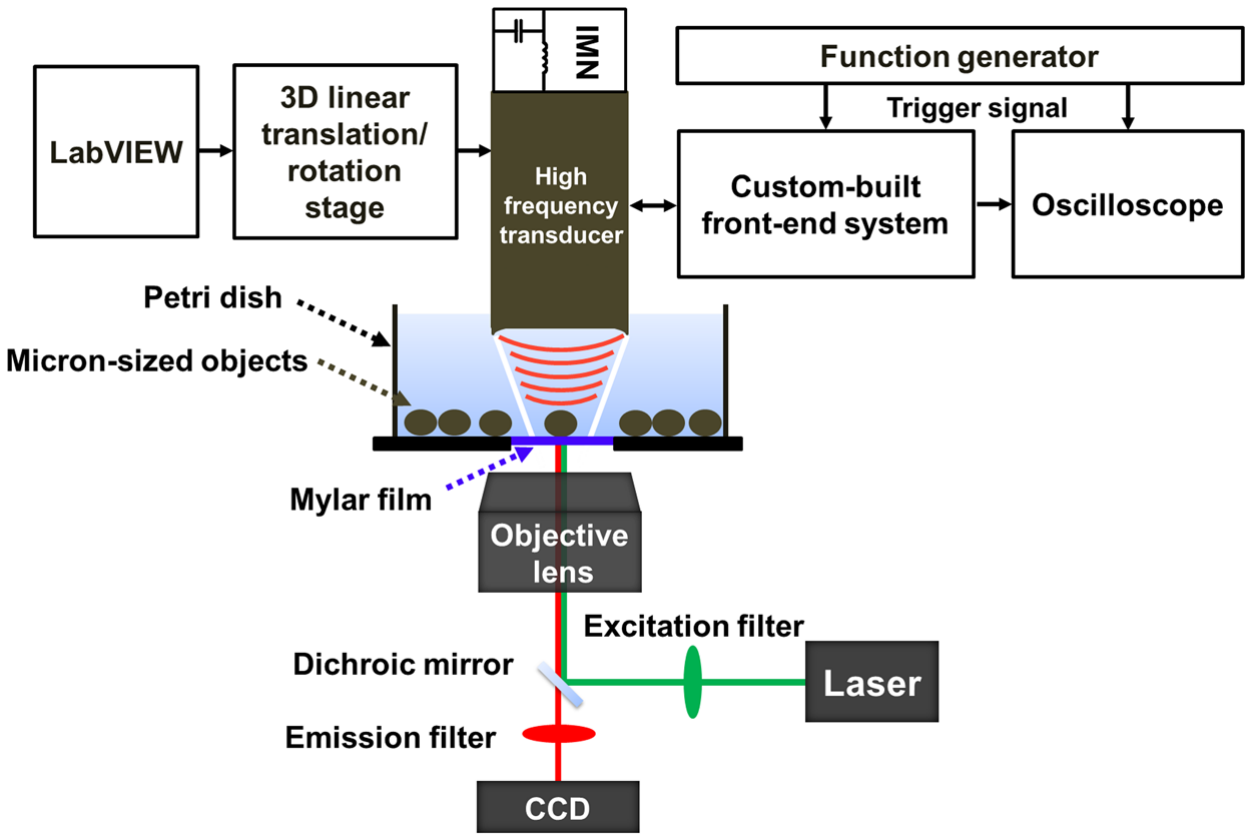
Schematic of developed label-free single-cell analysis system using high-frequency, high-PRF ultrasound microbeam. IMN stands for impedance matching network.

**Figure 2:**
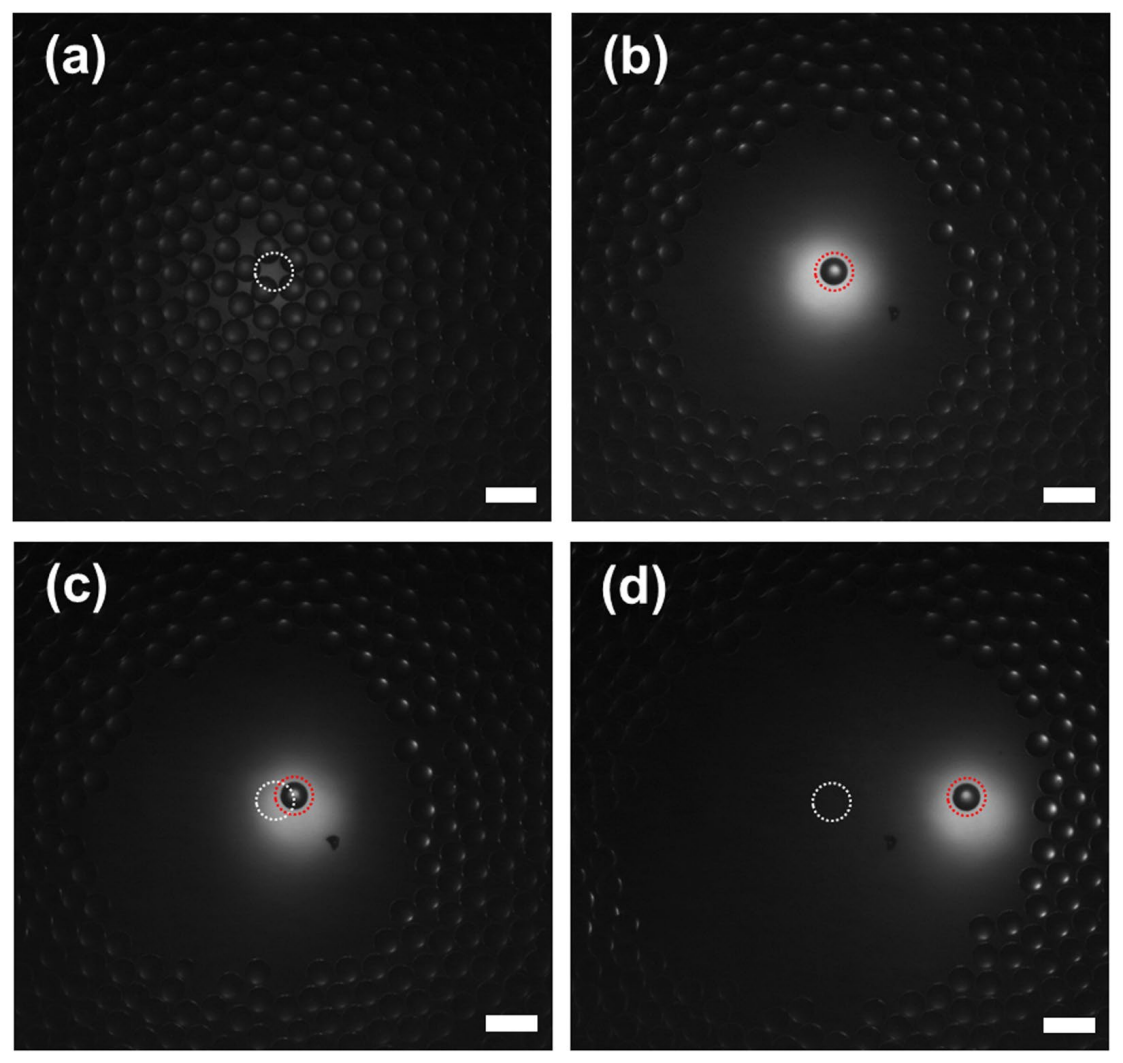
Images of polystyrene microspheres acquired by inverted fluorescence microscope (**a**) before and (**b**) after transmitting monocycle pulses with a length of 6.7 ns and a magnitude of 10 V at a PRF of 167 kHz to tightly focused high-frequency ultrasound transducer. As the transducer was moved to the right while delivering ultrasound microbeams at a PRF of 167 kHz, the captured microsphere was also moved in the same direction (**c**) and (**d**). White and red dashed circles indicate the initial and moved locations of the transducer, respectively. Scale bars in the images indicate 100 µm.

A conceptual diagram of the developed label-free single-cell analysis system is shown in Fig. 1 (see the Methods, Figs S1 and S2 for the detailed technical and performance information about the system). By using the system, the capability of short ultrasound pulses generated at high PRF to trap one polystyrene microsphere was ascertained. For this purpose, the front-end system excited the tightly focused high-frequency ultrasound transducer by monocycle electrical pulses with a length of 6.7 ns and a magnitude of 10 V at a PRF of 167 kHz. It should be noted that the pulse length is considerably shorter than the travel time (2.6 μs in this study) of the transmitted ultrasound between the transducer and microsphere, unlike the conventional acoustic tweezers using ultrasound microbeam. Once ultrasound microbeam was transmitted to capture one target microsphere, it was observed that the microspheres spread around the trapped microsphere (see Fig. 2(a) and (b)). As the transducer was moved to the right while delivering ultrasound microbeams at a PRF of 167 kHz, the captured microsphere was also moved in the same direction (Fig. 2(c) and (d)), which indicated that the short ultrasound pulses successfully trapped and separated one microsphere. The acoustic trapping force induced under the experimental conditions was measured using the micropipette aspiration method^37^; the value was 5.2 ± 0.43 nN (n = 4) at a distance of 1.5 μm from the centre of the ultrasound microbeam.

The effect of change in PRF on acoustic trapping force was also investigated using the monocycle electrical pulses with a length of 6.7 ns and a magnitude of 50 V, which were the same conditions for experiments on RBCs and PNT1A cells. To ascertain the effect, the acoustic trapping forces of ultrasound microbeams generated at PRFs of 33, 67, and 167 kHz were measured using the micropipette aspiration technique at a room temperature of 26 °C. The maximum trapping force was found to be 25.66 ± 2.81 nN, 75.35 ± 4.61 nN, and 122.44 ± 13.4 nN at PRFs of 33, 67, and 167 kHz, respectively (see the Supplement Information and Fig. S3). This result reveals that the acoustic trapping force increases with PRF.

### Size determination of trapped polystyrene microsphere

**Figure 3:**
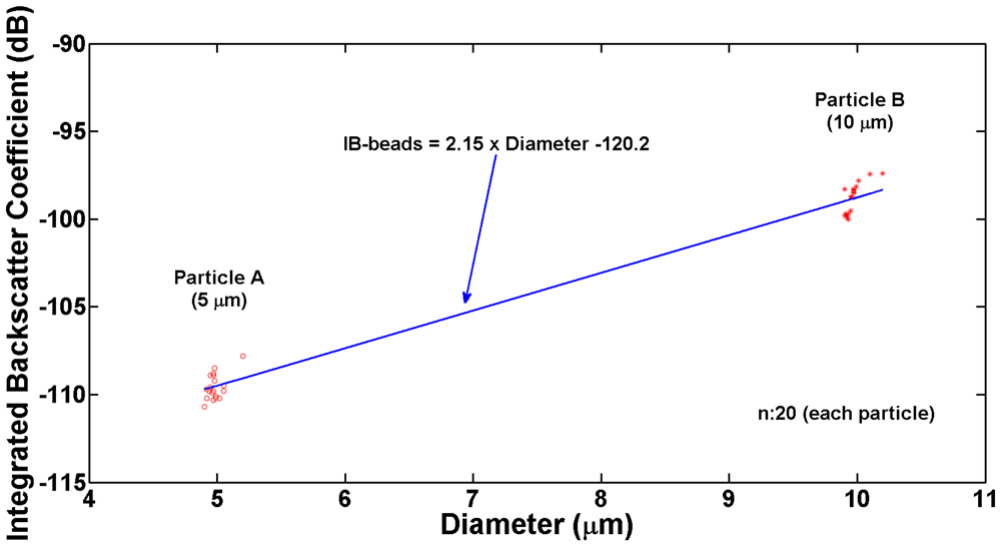
Measured integrated backscattering coefficients of trapped microspheres with sizes 5 and 10 μm in diameter. The number of each sample was 20. The blue solid line indicates the slope of the IB coefficient obtained by linear regression analysis.

To demonstrate the capability of the proposed method to capture one micro-sized particle and measure its physical properties simultaneously, we measured the IB coefficient of the trapped polystyrene microsphere because this coefficient is related to the size of a particle^38,39^. For this experiment, the monocycle pulses with a length of 6.7 ns and a magnitude of 50 V at a PRF of 167 kHz were used for simultaneous capture and measurement. The IB coefficients of 20 microspheres with a diameter of 5 μm and 20 microspheres with a diameter of 10 μm were −109.52 ± 0.75 dB and −98.84 ± 0.84 dB, respectively (Fig. 3). Note that the mean and standard deviation of the sizes of the two microsphere groups were 4.98 ± 0.06 μm and 9.97 ± 0.07 μm, respectively. The IB coefficients of the two different sized microspheres were statistically significant (p-value <0.01, 99% confidence interval for the difference between the two means (i.e., 10.68 dB): 10.03–11.33 dB). The IB coefficient slope of the microspheres, obtained by linear regression analysis, was approximately 2.15 dB/μm (99% confidence interval for the slope of the line of means: 2.02–2.27 dB/μm). This result reveals that small polystyrene microspheres exhibit lower IB coefficients than large ones, which is in good agreement with the previous studies^38^. Additionally, this indicates the possibility that the proposed system can facilitate the separation and identification of target cells in a heterogeneous cell population.

### Separation and differentiation of red blood cells and cancer cells

**Figure 4:**
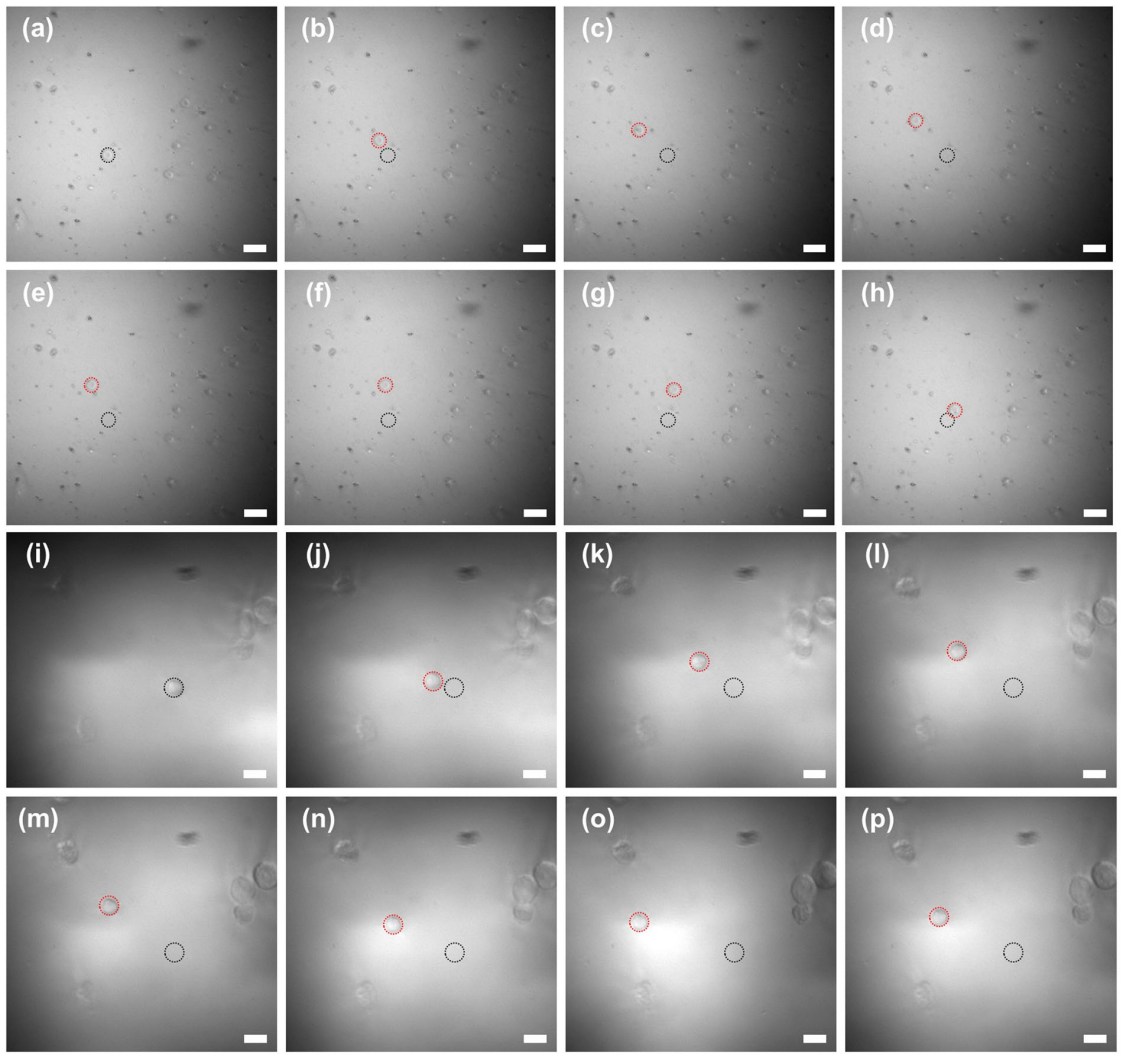
Inverted fluorescence microscope images of RBCs (**a**) to (**h**) and PNT1A cells (**i**) to (**p**) acquired after transmitting monocycle pulses with a length of 6.7 ns and a magnitude of 50 V at a PRF of 167 kHz to tightly focused high-frequency ultrasound transducer. The trapped cells were moved with the movement of the transducer; RBC movement is shown in (**b**) to (**h**) and PNT1A cell movement in (**j**) to (**p**). Black and red dashed circles indicate the initial and moved locations of the transducer, respectively. Scale bars in the images indicate 20 µm.

**Figure 5:**
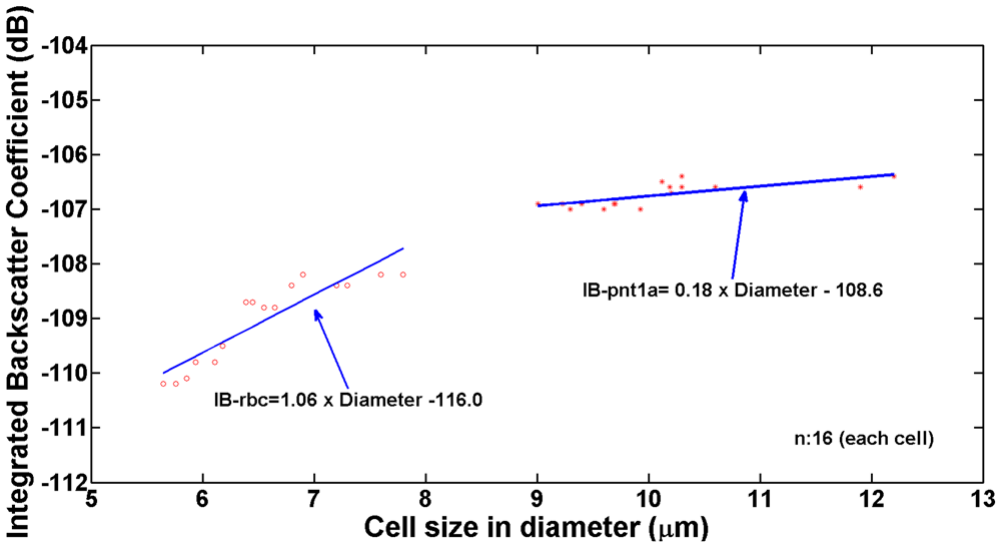
Measured integrated backscattering coefficients of trapped RBCs and PNT1A cells. The number of each sample was 16. The blue solid line indicates the slope of the IB coefficient obtained by linear regression analysis.

We also examined the possibility to utilize this approach to separate and differentiate RBCs and PNT1A cells. As shown in Fig. 4, the ultrasound microbeam generated by the monocycle pulses at a high PRF of 167 kHz was able to capture either one RBC or one PNT1A cell in the sample with the two types of cells and to move the target cell for separation; the initial and migration locations of the cell in response to the ultrasound microbeam movement are indicated by black and red dashed circles, respectively. While trapping the cell, the IB coefficients of RBCs with a diameter range of 6–8 μm and PNT1A cells of 9–12 μm were measured, as shown in Fig. 5; the mean and standard deviation of their sizes were 6.57 ± 0.66 μm and 10.10 ± 0.88 μm, respectively. The IB coefficients from 16 RBCs and 16 PNT1A cells were, respectively, −109.03 ± 0.77 dB and −106.74 ± 0.22 dB, which is statistically significant (p-value <0.01, 99% confidence interval for the difference between the two means (i.e., 2.29 dB): 1.77–2.79 dB). Additionally, the slope of IB coefficients of the RBCs and PNT1A cells, obtained by linear regression analysis, were approximately 1.06 dB/μm and 0.18 dB/μm (99% confidence intervals for the slope of the line of means: 0.67–1.45 dB/μm and 0.04–0.31 dB/μm), respectively. As a result, the proposed label-free single-cell analysis based on short acoustic microbeams enables us to separate one target single cell from a heterogeneous cell population and to simultaneously identify the cell by its physical characteristics such as the IB coefficient of the cell.

### Cell viability in the interaction between acoustic trapping force and cell

**Figure 6:**
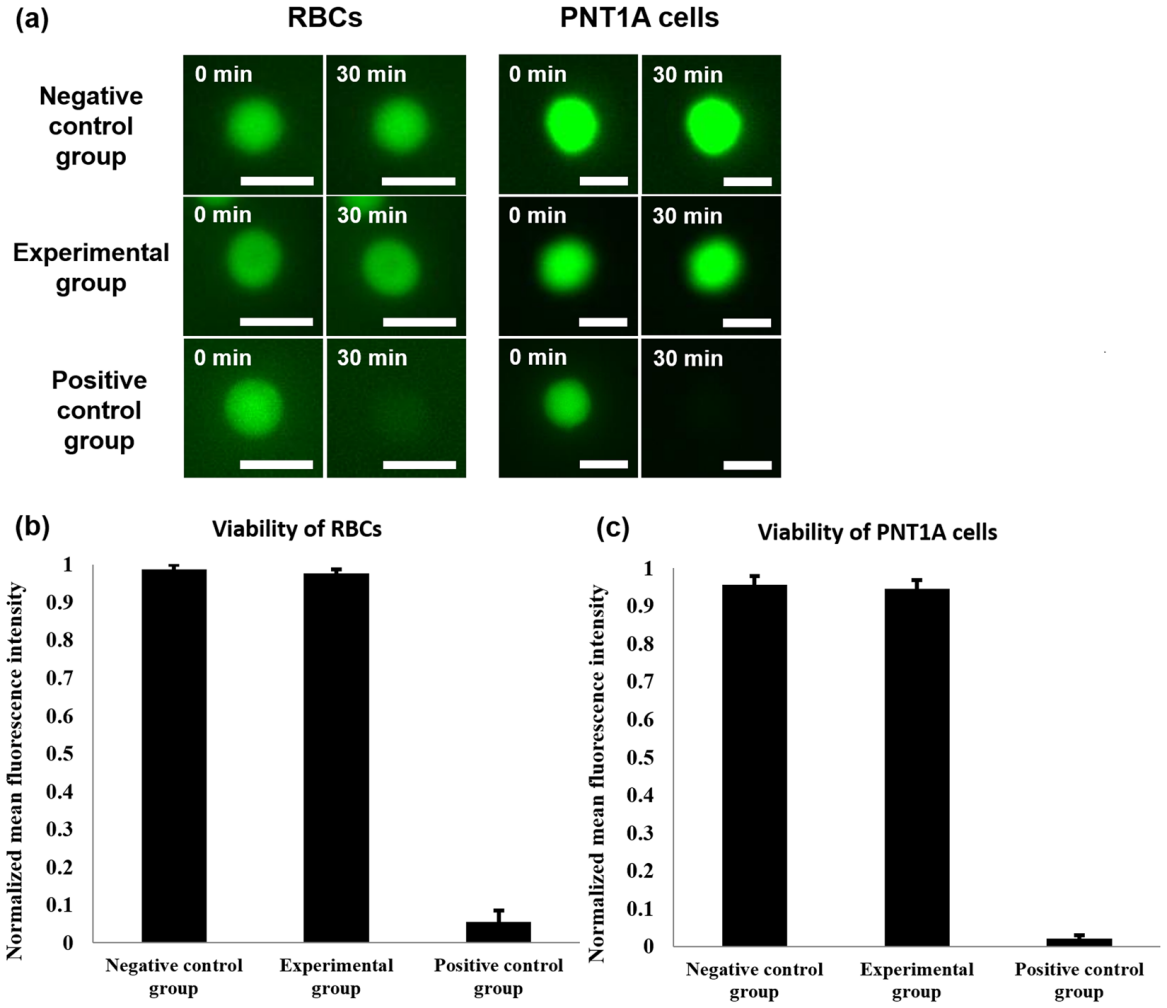
Results of the cell viability experiments: (**a**) Representative fluorescence images from the negative control, experimental, and positive control groups, which were acquired at 0 and 30 min. Scale bars in the images indicate 10 µm. Quantitative analysis of cell viability for (**b**) RBCs (p-value from the *t* test of the negative control versus experimental groups: 0.21 > 0.05, n = 20) and (**c**) PNT1A cells (p-value from the *t* test of the negative control versus experimental groups: 0.19 > 0.05, n = 20).

Cell viability test was performed to verify that our approach is safe to RBCs and PNT1A cells. For this purpose, the cell viability of the positive control, negative control, and experimental groups was investigated and compared. The RBCs and PNT1A cells emitted bright green fluorescence even after 30 min of delivering the monocycle pulses, which was similar to the negative control groups (see the first and second rows in Fig. 6(a)). The normalized mean fluorescence intensities from the RBCs and PNT1A cells were obtained by dividing the fluorescence intensity measured after 30 min by the initial intensity; the values obtained from the RBCs were 0.986 ± 0.012 in the case of the negative control group and 0.976 ± 0.01 in the experimental group. These from the PNT1A cells were 0.956 ± 0.023 and 0.946 ± 0.021 (Fig. 6(b) and (c)). Although a slight decrease in normalized mean fluorescence intensity was observed, there was no significant difference between before and after the acoustic trapping for 30 s; the p-value was 0.21 for 20 RBCs and 0.19 for 20 PNT1A cells. In contrast, very low green fluoresce was detected in the positive control cells at 30 min after treatment with 1% bleach (see the third row in Fig. 6(a)); the normalized mean fluorescence intensities from the RBCs and PNT1A cells were 0.054 ± 0.031 and 0.021 ± 0.009. The results of the cell viability test show that the high-frequency ultrasound microbeam generated under the proposed driving condition can manipulate single cells without compromising cell viability.

## Discussion

Conventional acoustic tweezer techniques employed long ultrasound pulses for holding either a particle or a single cell. Although this method is a useful tool for manipulating single cells with a relatively large trapping force and simple workflow compared to other approaches, separate procedures are still required for isolating and characterizing particular types of single cells. In this paper, we demonstrated that acoustic trapping force of short ultrasound pulses can be controlled by PRF; consequently, it is possible to capture a single cell and measure its IB coefficient simultaneously. Additionally, it has been confirmed that the ultrasound pulses used for separating RBCs and PNT1A cells and trapping one target cell are sufficiently short for determining its size based on the measured IB coefficient (Fig. 5); a size difference of 1.3 μm between the largest RBC (7.8 μm) and the smallest PNT1A cell (9.1 μm) were distinguished by an IB difference of 1.2 dB. Although it was demonstrated that the proposed label-free approach is capable of measuring the IB coefficient of a target cell under trapping as an example in this paper, we believe that this experimental result is a prominent stepping stone for the capture and the measurement of other physical properties of various cells such as the mechanical stiffness and shape of cancer cells^40,41^. Especially, the different slopes of the IB coefficient of RBCs and PNT1A cells shown in Fig. 5 indicate another possibility of measuring the mass density and compressibility of cells after separation; these are the secondary factors influencing the IB coefficient of cells. Since RBCs in mammals do not have a cell nucleus that is regarded as a densely packed object than the surrounding cytoplasm, an IB coefficient is likely to be different between RBCs and cancer cells even if the two types of cells have similar sizes. It should be noted that the slope of the regression curve for the RBCs in Fig. 5 was about five times steeper than for the PNT1A cells and two times less steep than for the polystyrene beads. This result may be explained by the difference in the compressibility and density of those samples, which will be intensively investigated in the near future.

The proposed label-free single-cell analysis is possible due to the front-end system developed to capture one particle and measure its IB coefficient simultaneously. The development of a front-end system capable of generating considerably high-frequency (>100 MHz), high-amplitude ultrasound short pulses at an adjustable PRF for cell trapping and receiving considerably small backscattering signals from one particle was challenging; commercial systems with these capabilities are not currently available in the market. To achieve the desired level of acoustic pressure, we employed the electrical impedance matching method developed and reported previously^42^ that makes it possible to efficiently convert the electrical voltage applied to the developed high-frequency ultrasound transducer into acoustic pressure. Other notable features are the capabilities to generate ultrasound microbeams at high PRF (up to 1 MHz) without compromising the output voltage level and to enhance the quality of considerably small backscattering signals in the signal amplifier with a considerably low noise figure of 1.6 dB. The developed system for a single element transducer can be used to extend to an ultrasound array-based system with 5 × 5 or 10 × 10 elements to increase throughput.

In Fig. 2, it was observed that the polystyrene particles were spread around the trapped particle because of interaction between scattering and gradient forces^43,44^. The distance between the trapped particle and surrounding particles is linearly proportional to acoustic trapping force, which is related to the capability to separate one target cell from heterogeneous cells by the trapping force. Although the relatively high amplitude of ultrasound microbeams at high PRF did not cause the cell viability to be impaired (Fig. 6), this may result in degrading the performance of high-frequency ultrasound transducers. For relatively high-density cells, additionally, an increase in acoustic trapping force may be required for more reliable cell separation. If a desired level of acoustic trapping force cannot be reached by increasing PRF, another capture and interrogation strategy is possible; initial cell separation is conducted using the conventional long ultrasound pulses at a certain PRF, and interrogation under trapping is subsequently performed using the proposed short ultrasound pulses at the same or higher PRF. This is based on the fact that acoustic trapping force is determined by pulse amplitude, pulse length, and PRF. For example, the acoustic trapping force produced by short electrical pulses with a length of 6.7 ns and a magnitude of 10 V at a PRF of 167 kHz was similar to that by long pulses with a length of 1 μs and a magnitude of 3.8 V at a PRF of 1 kHz.

Like optical tweezers, acoustic tweezers may lead to an increase in local temperature in a measurement chamber. To predict the effect of the proposed method on temperature rise, we calculated the maximum temperature increase under the experimental condition by using a mathematical model expressed as^45,46^

1 $${\rm{\Delta }}{T}_{max}=\frac{2\cdot \alpha \cdot {I}_{TA}\cdot {\rm{\Delta }}t}{{C}_{v}},$$

where $\alpha$ = 10.04 dB/cm at 153 MHz, I_TA_ (The ultrasonic temporal average intensity) = 0.0062 W/cm^2^, ${\rm{\Delta }}t$ = 1 s, *C*_*v*_ = 4.18 J/cm^3^ °C in our experimental condition. From (1), ${\rm{\Delta }}{T}_{max}$ was calculated to be 0.029 °C that is negligible. Therefore, we concluded that the adverse thermal effect is not the case in our approach.

Acoustic trapping with tightly focused ultrasound microbeam is possible owing to a change in the momentum of incident beams in the interaction between a gradient force and a scattering force^43,44^. Trapping performance is stable when the gradient force pulling a particle toward beam focus exceeds the scattering force pushing the particle away from the focus in the direction of the incident beam. To trap a particle, theoretically, the wavelength of incident ultrasound beam (λ) should be larger than the diameter of the particle (D) (i.e. D > λ)^43^, and this was experimentally verified^47,48^. However, it was also ascertained that ultrasound microbeam can be used for trapping a particle even when D < λ^49,50^ and D ≈ λ^51,52^. This study was conducted using an ultrasound microbeam of 10 μm wavelength and particles of various sizes (i.e. microspheres with diameters of 5 and 10 μm, RBCs with diameters in the range of 6–8 μm, and PNT1A cells with diameters in the range of 9–11 μm) and showed that particles of various sizes can be trapped as in previous studies. However, the theoretical background of why acoustic trapping is possible in the case of D < λ and D ≈ λ is still not investigated and should be established and verified in the near future.

## Methods

### Label-free single-cell analysis system using high-frequency, high-PRF ultrasound microbeam

The proposed label-free single-cell analysis begins with the capture of one target cell by high-frequency, high-PRF ultrasound microbeam. A tightly focused ultrasound transducer was developed for generating high-frequency microbeam. In pulse–echo and wire target imaging tests, it was found that the transducer exhibits a centre frequency of 153 MHz, a −6 dB fractional bandwidth of 12% (144–162 MHz), and axial and lateral beam widths of 28.5 and 8.6 μm at a focal length of 1.95 mm (Fig. S1). Furthermore, a front-end system was designed and implemented for generating high-frequency, high-PRF ultrasound microbeam. The developed front-end system is able to generate monocycle bipolar pulses with a centre frequency of 200 MHz, a −6 dB fractional bandwidth of 90% (110–290 MHz), a maximum peak-to-peak amplitude of 50 V, a maximum PRF of 1 MHz (Fig. S2). As shown in Fig. 1, the developed impedance matching network (IMN) was used for connecting the transducer and the front-end system to efficiently deliver electronic signals from the system to the transducer, and vice versa. After capturing one target cell, an inverted fluorescence microscope with a 10X objective lens (IX71, Olympus, Center Valley, PA, USA) and an image acquisition and analysis tool (Metamorph, Molecular Devices, Sunnyvale, CA, USA) were used for visual confirmation of the capture and the movement of the trapped cell; time-resolved bright-field images were acquired as the location of the microbeam was changed. As the custom-built front-end system is able to excite the high-frequency ultrasound transducer by monocycle bipolar pulses at a high PRF of 167 kHz, it is possible to trap one single target cell and to measure the IB coefficient of the trapped cell simultaneously. Note that the echo received after transmitting one ultrasound pulse was used to measure an IB coefficient after capturing the target cell although the average of multiple echoes can be used to improve a signal-to-noise ratio. Detailed technical and performance information about the label-free single-cell analysis system can be found in the Supplementary Information.

### Cell preparation

Fresh human whole blood from a volunteer was obtained with informed consent. The blood-gathering and all experiments with the obtained blood were conducted in accordance with the guidelines and regulations approved by the institutional review board (IRB) of the University of Southern California (UP-16-00713). Whole blood was centrifuged with phosphate-buffered saline (PBS) at 500 $\times$ g for 10 min to separate red blood cells (RBCs) from white blood cells (WBCs), platelets, blood plasma, and PBS (Thermo Fisher Scientific, Waltham, MA). After gently eliminating the supernatant, the RBCs were resuspended with PBS, and then the cells with PBS were once again centrifuged and resuspended with a mixed solution of PBS and Alsever’s solution. Normal SV40 immortalized epithelial prostate (PNT1A) cells were cultured in RPMI 1640 supplemented with 10% fetal bovine serum (FBS) and incubated in a humidified 5% CO_2_ at 37 °C incubator. The PNT1A cells were gently washed twice with PBS and dispensed with the TrypLE solution in the incubator for 5 min, and centrifuged at 150 $\times$ g for 5 min. After gently eliminating the supernatant, PNT1A cells were resuspended with PBS, and then the cells with PBS were once again centrifuged and resuspended with the dulbecco’s phosphate-buffered saline (DPBS) with Ca^2+^ (Thermo Fisher Scientific, Waltham, MA).

Calculation of integrated backscattering coefficients. An IB coefficient is defined as the ratio of backscattered ultrasound energy from a scatterer volume to the one from a flat quartz target. This is expressed as^39^

2 $${\rm{IB}}=10\cdot {lo}{{g}}_{10}({\int }_{fc-{\rm{\Delta }}f}^{fc+{\rm{\Delta }}f}\frac{|V(f){|}^{2}}{|R(f){|}^{2}}df),$$

where R(f) and V(f) are, respectively, the frequency spectrum of the recorded backscattered signals from the quartz target and the trapped objects (particles or cells). Moreover, ${f}_{c}$ and $\triangle f$ represent the centre frequency and the −6 dB bandwidth of R(f), respectively. The pulse–echo response of the high-frequency transducer to a flat quartz target was recorded using an oscilloscope (104MXi, LeCroy, Santa Clara, CA) at 10 GHz sampling rate; the flat quartz target was immersed in a degassed deionized water container. For V(f), the same oscilloscope was used to record the backscattered signal from the captured object on the acoustically transparent Mylar film in the chamber filled with Alsever’s solution with phosphate-buffered saline (PBS). Note that the calibration of acoustic trapping force was also investigated in the same chamber. Fourier transform of the recorded backscattered signals and calculation of IB coefficients were performed on a MATLAB program (MathWorks Inc., Natick, MA) after the experiments.

### Cell viability study

To examine cell viability, RBCs and PNT1A cells were washed twice with PBS and stained with a membrane-permeable live-cell labelling dye (Calcein, AM, Thermo Fisher Scientific, Waltham, MA). Detailed staining process can be found in the previously reported paper^50^. For the negative control values, 20 RBCs and 20 PNT1A cells were stained and live-cell fluorescence images were acquired after 30 min. On the other hand, each of 20 RBCs and 20 PNT1A cells was trapped by the short ultrasound pulses with a length of 6.7 ns and a magnitude of 50 V at a PRF of 167 kHz for 30 s after being stained. For the positive control values, 20 RBCs and 20 PNT1A cells were treated with 1% bleach for 30 min after being stained with Calcein dye. At 30 min after this experiment, live-cell fluorescence images were obtained and the normalized mean fluorescence intensity was computed from the images.

### Statistical analysis

Statistical analysis was conducted using a two-tailed paired t-test. The measured values were expressed as mean ± standard deviation.

## Electronic supplementary material

Supplementary Information
