# Supplementary material for: Label-free analysis of the characteristics of a single cell trapped by acoustic tweezers
Source: Sci Rep. 2017 Oct 26;7:14092. doi: 10.1038/s41598-017-14572-w (PMC5658370; doi:10.1038/s41598-017-14572-w)
Supplement: Supplementary file 1 — Supplementary Information [file 41598_2017_14572_MOESM1_ESM.pdf]

Supplementary Information

Label-free analysis of the characteristics of a single cell trapped by acoustic tweezers

Min Gon Kim[1†], Jinhyoung Park[2†], Hae Gyun Lim[1], Sangpil Yoon[1], Changyang Lee[3], Jin Ho Chang[3,4]*, and K. Kirk Shung[1]

[1]Department of Biomedical Engineering, University of Southern California, Los Angeles, CA, 90089, USA
[2]Department of Biomedical Engineering, Sungkyunkwan University, Suwon, Gyeonggi-do, 16419, Republic of Korea
[3]Department of Biomedical Engineering, Sogang University, Seoul 04107, Republic of Korea
[4]Department of Electronic Engineering, Sogang University, Seoul, 04107, Republic of Korea

*jhchang@sogang.ac.kr

Label-free cell analysing system. This system comprises a high-frequency ultrasound transducer, front-end system, impedance matching network (IMN), and system control module, as shown in Fig. 1. The tightly focused high-frequency transducer was designed and fabricated using lithium niobate ($LiNbO_3$). The fabrication process can be found in detail in the previously reported paper [1]. The aperture size and F-number of the transducer were 2.6 mm and 0.75, respectively. The IMN was developed for enhancing energy transfer efficiency from the front-end system to the ultrasound transducer. The performance of IMN proved to be suitable for the application to label-free cell analysis and its detailed configuration can be found in *Kim et al.* [2]. The inverted fluorescence microscope (IX71, Olympus, Center Valley, PA) was used for acquiring time-resolved bright-field images for visual confirmation of trapping a particle and moving along the direction of the high-frequency ultrasound microbeam. A three-dimensional linear translation/rotation stage controlled by a customized LabVIEW (National Instruments, Austin, TX) program was used for precisely controlling the location of the transducer with IMN during the experiments. The custom-built front-end system and an oscilloscope were responsible for exciting the transducer to generate ultrasound microbeams and for receiving backscattered signals from a trapped single object. The front-end system developed for this study comprises a transmitter, protection circuits, and a receiver. In the transmitter, monocycle bipolar pulses with an amplitude of 2 $V_{p\text{-}p}$ are generated at a maximum pulse repetition frequency

(PRF) of 1 MHz. These pulses are amplified to 50 $V_{p\text{–}p}$ in the power laterally diffused metal oxide semiconductor (LDMOS) transistor [3]. Moreover, a diode-based expander and limiter were used for the protection of the transmitter and receiver circuitry [4],[5]. The expander blocks the echo signals from the ultrasound transducer to the transmitter, and the limiter blocks the high voltage generated in the transmitter to the receiver. The primary part of the receiver is a low noise amplifier (LNA) that enhances a signal-to-noise ratio (SNR).

Performance evaluation of tightly focused high-frequency ultrasound transducer and front-end system developed for this study. To examine the performance of an ultrasound transducer, a pulse–echo response and an ultrasound image of a 2.5 μm diameter tungsten wire were acquired (Fig. S1). For this, a flat quartz target or a tungsten wire was immersed into a degassed deionized water container. The position of the transducer integrated with IMN was adjusted for locating the target at the focal length of the transducer, which was controlled by a 3D linear translation/rotation stage (ILS100HA, Newport, Irvine, CA) with a customized MATLAB program (MathWorks Inc., Natick, MA). The transducer was excited with an electrical pulse of energy 1 μJ, and the echoes were amplified by 20 dB before digitized by a 12-bit analog-to-digital card (CS122G1, Dynamics Signals LLC., Lockport, IL) at a sampling frequency of 1 GHz. From the pulse–echo response, the centre frequency and −6 dB bandwidth of the transducer were found to be 153 MHz and 12% (144–162 MHz), respectively. Moreover, it was found from the wire target image that its axial and lateral resolutions were 28.5 and 8.6 μm, respectively. The performance evaluation of the developed high-frequency ultrasound front-end system was conducted. For this purpose, electrical pulses at the output of the developed pulse generator were measured using a digital phosphor oscilloscope (TDS 5052, Tektronix, Beaverton, Oregon), which were generated at a PRF of 167 kHz (Fig. S2). In this experiment, it was found that monocycle bipolar pulses generated by the front-end system had a centre frequency of 200 MHz, a −6 dB bandwidth of 90% (110 MHz–290 MHz), and a maximum peak-to-peak amplitude of 50 V. Additionally, the pulses were successfully generated at a PRF of 167 kHz. The amplification gain of the receiver in the front-end system was 19.5 dB and its noise figure was measured to be 1.6 dB by using a spectrum analyser (E4402B, Agilent technologies, Santa Clara, CA).

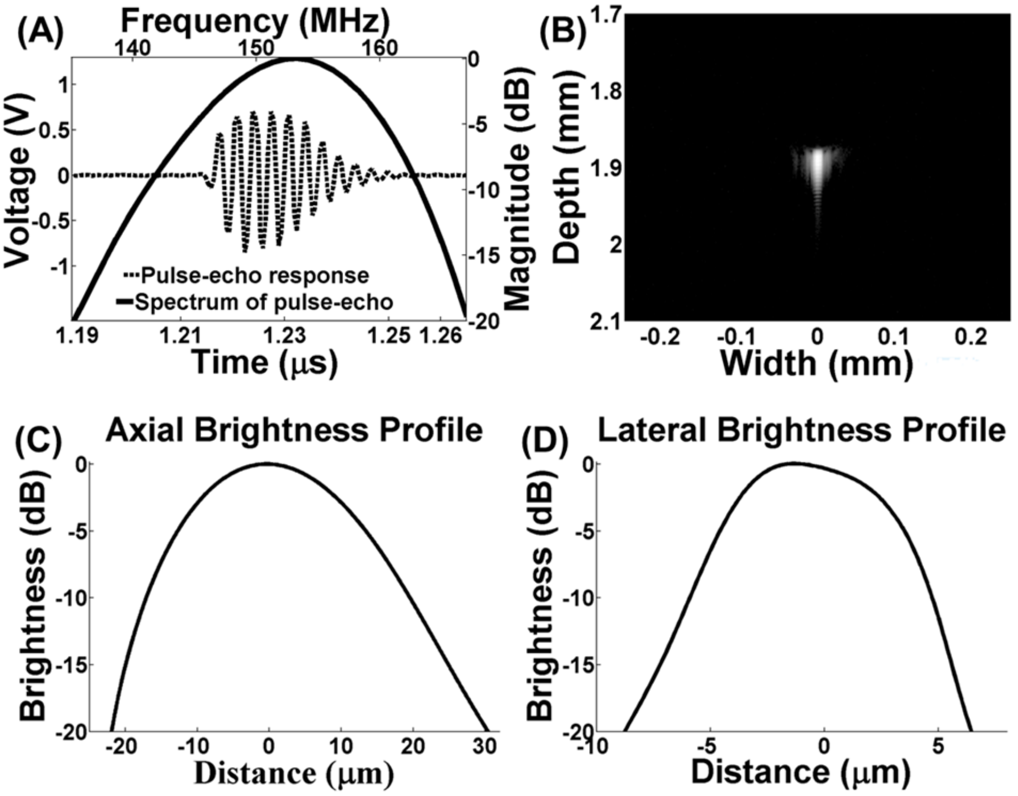

Fig. S1. Performance of fabricated ultrasonic transducer with impedance matching network (IMN). (A) Pulse–echo waveform (dotted line) of the ultrasonic transducer with IMN and its frequency spectrum (solid line). The centre frequency and −6 dB bandwidth measured 153 MHz and 12% (144–162 MHz), respectively. (B) B-mode image of a 2.5 μm diameter tungsten wire was acquired to examine the axial (C) and lateral (D) beam profiles. Axial and lateral resolutions were 28.5 and 8.6 μm, respectively.

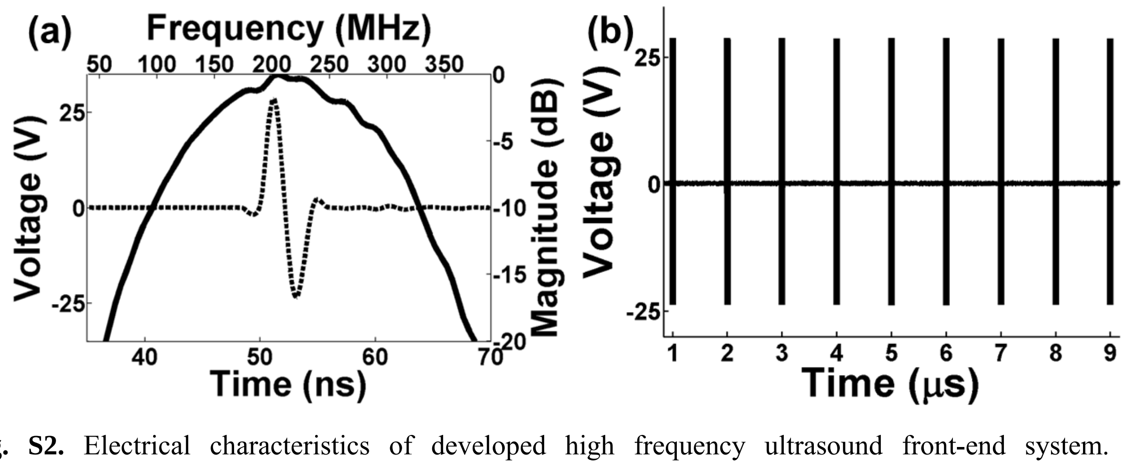

Fig. S2. Electrical characteristics of developed high frequency ultrasound front-end system. (A) Monocycle bipolar pulse (dotted line) with a maximum amplitude of 50 $V_{p\text{–}p}$ and its frequency spectrum (solid line): a centre frequency of 200 MHz and a −6 dB bandwidth of 90% (110–290 MHz). (B) Measured pulse train with a pulse repetition frequency of 1 MHz.

Change in acoustical trapping force as a function of pulse repetition frequency (PRF). Trapping forces from the acoustic tweezers were measured using a micropipette aspiration technique (MAT) [6]. The inner diameter of a micropipette was fabricated to be 3 μm by using a vertical micropipette puller (PC-10, Narishige, NY). The suction force was controlled by the control software (NBSC Controller, Neo biosystem, CA). One microsphere in the chamber filled with PBS was trapped by the respective ultrasound microbeams generated at PRFs of 33, 67, and 167 kHz. Note that the IB coefficient measurement was conducted under the same experimental conditions as the trapping force measurement. The suction force from a micropipette acting on the microsphere was equally balanced by the trapping force generated from the ultrasound microbeams. The microsphere gradually moved away from the centre of acoustic beam with increase in the suction force. Depending on the displacement of the trapped microsphere from the centre of ultrasonic beam area while trapping, the trapping force changes similar to an elastic spring [7]. At the fixed excitation voltages of 50 $V_{p\text{-}p}$, the maximum trapping force was found to be 25.66 ± 2.81, 75.35 ± 4.61, and 122.44 ± 13.4 nN at PRFs of 33, 67, and 167 kHz, respectively.

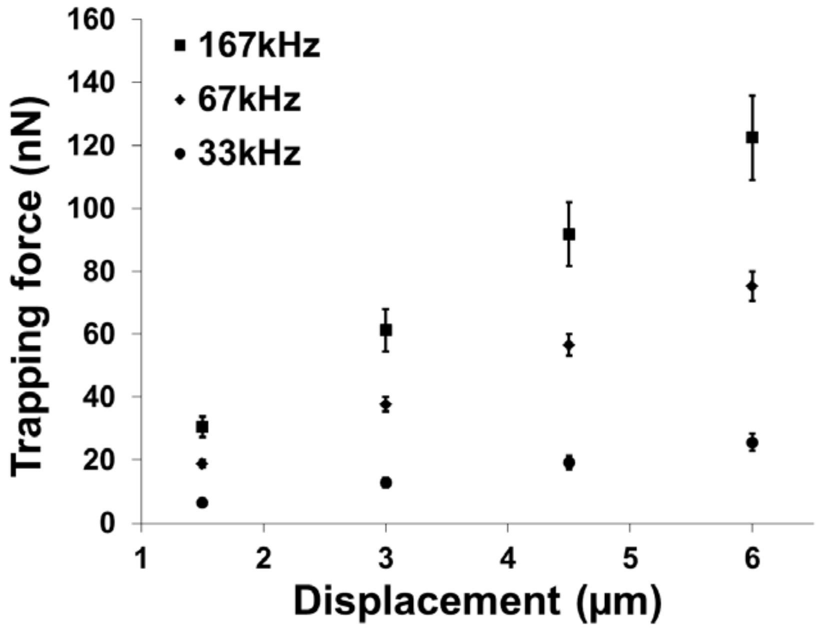

Fig. S3. Measurement of change in acoustic trapping force as a function of pulse repetition frequency (PRF).

References

1. Lam, K. H. *et al*. Development of lead-free single-element ultrahigh frequency (170–320 MHz) ultrasonic transducers. *Ultrasonics* **53**, 1033–1038 (2013).
2. Kim, M. G., Yoon, S., Kim, H. H. & Shung, K. K. Impedance matching network for high frequency ultrasonic transducer for cellular applications. *Ultrasonics* **65**, 258–267 (2016).
3. Kim, M. G., Choi, H., Kim, H. H. & Shung K. K. Bipolar pulse generator for very high frequency (>100 MHz) ultrasound applications. *Ultrason. Sympos. (IUS), IEEE Int.* 1567–1570 (2013).
4. Choi, H., Kim, M.G. & Shung, K. K. New MOSFET-based expander for high frequency ultrasound systems. *Ultrason. Sympos. (IUS), IEEE Int.* 623–626 (2012).
5. Choi, H., Kim, M.G., Cummins, T. M., Hwang, J. Y. & Shung, K. K. Power MOSFET-diode-based limiter for high-frequency ultrasound systems. *Ultrason. Imag.* **36,** 317–330 (2014).
6. Lim, H. G. *et al.* Calibration of trapping force on cell-size objects from ultra-high frequency single beam acoustic tweezer. *IEEE Trans. Ultrason. Ferroelectr. Freq. Control* **63**(11), 1988–1995 (2016).
7. Lee, J., Jeong, J. S. & Shung, K. K. Microfluidic acoustic trapping force and stiffness measurement using viscous drag effect. *Ultrasonics* **53**(1), 249–254 (2013).
